# Serum Pancreatic Stone Protein Across the Spectrum of Hypertensive Disorders of Pregnancy: Discrimination of Preeclampsia Compared with Conventional Inflammatory Indices

**DOI:** 10.3390/medicina62071361

**Published:** 2026-07-15

**Authors:** Sait Erbey, Mehmet Alican Sapmaz, Ömer Osman Eroğlu, Aziz Kından, Murat Polat, Bilge Erbey, İnci Kahyaoğlu

**Affiliations:** 1 Department of Obstetrics and Gynecology, Ankara Etlik City Hospital, 06170 Ankara, Türkiye; dr.alicansapmaz@hotmail.com (M.A.S.); omerosmaneroglu@gmail.com (Ö.O.E.); dr.muratpolat@hotmail.com (M.P.); mdincikahyaoglu@gmail.com (İ.K.); 2 Department of Obstetrics and Gynecology, Division of Perinatology, Ankara Etlik City Hospital, 06170 Ankara, Türkiye; azizkindan@hotmail.com; 3 Independent Obstetrics and Gynecology Specialist, 06800 Ankara, Türkiye; blgegoktas@gmail.com

**Keywords:** pancreatic stone protein, preeclampsia, gestational hypertension, biomarker, systemic inflammation, ROC analysis, hypertensive disorders of pregnancy

## Abstract

*Background and Objectives*: Hypertensive disorders of pregnancy (HDP), including gestational hypertension (GHT) and preeclampsia (PE), share clinical features but differ in pathophysiology and risk profile. Pancreatic stone protein (PSP), a pancreas-derived acute-phase glycoprotein, has emerged as a biomarker of systemic stress. We investigated whether PSP could discriminate PE from both GHT and normotensive pregnancy, and whether this discrimination extends beyond the discriminatory information captured by conventional CBC-derived inflammatory indices. *Materials and Methods*: In this prospective case–control study, 84 pregnant women were enrolled across three groups: normotensive controls (*n* = 42), GHT (*n* = 20), and PE (*n* = 22). Serum PSP was measured by ELISA. Seven CBC-derived inflammatory indices (NLR, PLR, MLR, SII, SIRI, PIV, AISI) were calculated. Three-group comparisons used Kruskal–Wallis or ANOVA with appropriate post hoc testing. Receiver operating characteristic (ROC) analysis was performed across multiple clinical scenarios. Binary and multinomial logistic regression analyses were conducted with control as the reference category. *Results*: Median serum PSP levels showed a progressive elevation across the HDP spectrum: 6.91 (5.47–9.73), 9.08 (8.29–10.36), and 11.07 (10.03–11.83) ng/mL for control, GHT, and PE groups, respectively (Kruskal–Wallis *p* < 0.001). All three pairwise comparisons remained significant after Bonferroni correction (control vs. GHT, *p* = 0.035; control vs. PE, *p* < 0.001; GHT vs. PE, *p* = 0.002). PSP showed moderate-to-good exploratory discriminatory performance for PE versus control (AUC 0.83; sensitivity 95.5%, specificity 66.7% at 8.61 ng/mL), PE versus GHT (AUC 0.80; sensitivity 68.2%, specificity 85.0% at 10.70 ng/mL), and PE versus all non-PE participants (AUC 0.82). None of the inflammatory indices reached statistical significance (all *p* > 0.05), although several (MLR *p* = 0.072; SIRI *p* = 0.071) showed borderline upward trends consistent with the severity gradient. Multinomial logistic regression showed a consistent graded association: each 1 ng/mL increase in PSP was associated with a 42% increase in the odds of GHT (vs. control) (OR 1.42; 95% CI: 1.05–1.92; *p* = 0.022) and approximately threefold higher odds of PE (vs. control) (OR 2.99; 95% CI: 1.57–5.68; *p* = 0.001). *Conclusions*: Serum PSP demonstrated a graded increase across the HDP spectrum and showed moderate-to-good discriminatory performance for PE, including differentiation from GHT. These findings suggest that PSP may capture biological information not reflected by conventional CBC-derived inflammatory indices. However, the proposed cutoffs should be regarded as exploratory, and larger multicenter studies with gestational-age-matched controls and direct comparison with angiogenic biomarkers are required before clinical implementation.

## 1. Introduction

Preeclampsia (PE) is a multisystem hypertensive disorder of pregnancy, defined by the new onset of hypertension (≥140/90 mmHg) accompanied by proteinuria or signs of end-organ dysfunction after 20 weeks of gestation in previously normotensive women. It represents one of the leading causes of maternal and perinatal morbidity and mortality worldwide, accounting for approximately 14% of maternal deaths globally and complicating 2–8% of all pregnancies [1,2,3]. A recent meta-analysis estimated a global PE prevalence of 4.43%, with regional variation reflecting differences in healthcare capacity and diagnostic criteria [4,5,6].

Within the broader category of hypertensive disorders of pregnancy (HDP), gestational hypertension (GHT)—defined as new-onset hypertension after 20 weeks of gestation without proteinuria or end-organ dysfunction—has historically been viewed as a milder phenotype. However, contemporary evidence indicates that GHT and PE may represent distinct points along a shared pathophysiological continuum, with up to 25% of GHT cases progressing to PE and both conditions sharing endothelial dysfunction and angiogenic imbalance as core mechanisms [7,8,9]. The clinical and prognostic distinction between GHT and PE therefore represents an important diagnostic challenge, and biomarkers that reliably differentiate the two could refine risk stratification, surveillance intensity, and treatment decisions.

Despite decades of research, the precise pathophysiological cascade underlying PE remains incompletely elucidated. The prevailing two-stage model posits that aberrant trophoblast invasion leads to incomplete spiral artery remodeling, placental ischemia, and release of soluble antiangiogenic factors—primarily soluble fms-like tyrosine kinase-1 (sFlt-1) and soluble endoglin (sEng)—into the maternal circulation, culminating in endothelial dysfunction, oxidative stress, and systemic inflammation [2,10].

The inflammatory component of PE is well-recognized. Excessive reactive oxygen species (ROS) generated by hypoxia–reoxygenation injury of the placenta activate the NF-κB pathway, triggering release of proinflammatory cytokines including IL-6, TNF-α, and IL-1β, and promoting leukocyte–endothelial adhesion via upregulation of ICAM-1 and VCAM-1 [11]. Mitochondrial dysfunction within fetal trophoblast cells exacerbates this oxidative-inflammatory cycle, reinforcing placental dysfunction [11]. However, the systemic inflammatory response in PE is qualitatively distinct: it is neither as quantitatively marked nor as broadly dysregulated as in conditions such as sepsis or classical systemic inflammatory response syndrome.

Hematological indices derived from complete blood count parameters—NLR, PLR, MLR, SII, SIRI, PIV, and AISI—have been proposed as inexpensive surrogates of systemic inflammation. Their performance in PE is, however, inconsistent across studies. In a large retrospective cohort, Kong et al. demonstrated that first-trimester immune markers including SII and AISI were independently associated with HDP risk in a linear dose–response manner, with distinct biomarker profiles between gestational hypertension and preeclampsia subtypes [12]. Earlier work by Kirbas et al. similarly reported significantly elevated first-trimester NLR values in women who subsequently developed preeclampsia compared with healthy controls [13], while Maziashvili et al. reported significant differences in age-stratified PLR and SII values between preeclamptic and healthy pregnancies [14]. Conversely, peripartum analyses of established PE have demonstrated only modest or non-significant elevations in these indices compared with normotensive controls [15]. This heterogeneity suggests that non-specific inflammatory indices may reflect early immunological priming rather than PE-specific pathology at the time of clinical presentation.

Pancreatic stone protein (PSP), also known as lithostathine or regenerating protein 1α (REG1A), is a 14 kDa C-type lectin glycoprotein of 144 amino acids encoded by the REG gene family and secreted predominantly by pancreatic acinar and beta cells [16,17]. PSP has been established as a sensitive biomarker for bacterial infection and sepsis, demonstrating superior performance compared to CRP and procalcitonin in meta-analytic assessments [18]. Beyond infection, PSP is elevated in diabetic kidney disease, ARDS, and conditions of systemic stress—consistent with its proposed role as an acute-phase protein secreted by the pancreas in response to remote organ injury and immune activation. Its release is thought to reflect systemic inflammatory and cellular-stress signaling reaching the pancreas rather than a pancreas-specific process, and the molecular mechanisms potentially involved are considered, as hypotheses, in the Discussion [17,19].

PSP has recently been studied in the context of pregnancy-related diseases. Vonzun et al. established reference values for PSP in healthy pregnant women at 7.9 ± 2.6 ng/mL for singleton gestations, noting significantly higher values in multiple pregnancies and a modest increase across trimesters [20]. Brun et al. reported significantly elevated PSP levels in pregnant women with preeclampsia and HELLP syndrome, while no significant elevation was observed in PPROM or COVID-19-positive pregnancies, suggesting a relatively specific association of PSP with hypertensive and severe placental-organ dysfunction syndromes rather than non-specific pregnancy inflammation [21].

A critical unresolved question is whether PSP elevation in PE is specific to PE pathophysiology or merely reflects a generic systemic inflammatory response also captured by routinely available CBC-derived indices. Furthermore, no study has yet examined whether PSP can discriminate PE from GHT—a clinically essential distinction within the HDP spectrum. If PSP elevation tracks the disease-severity gradient more sensitively than standard inflammatory indices, and if PSP further differentiates PE from GHT, this would support the hypothesis that PSP captures discriminatory information not fully resolved by conventional CBC-derived markers, motivating further evaluation of its potential utility as an adjunctive biomarker.

Although the sFlt-1/PlGF ratio represents the most extensively validated angiogenic biomarker for PE risk stratification, its widespread implementation remains constrained by assay cost, dependence on dedicated immunoassay platforms, and limited availability in many secondary-care obstetric settings—particularly outside high-resource centers. These access-related considerations reinforce the value of investigating alternative or complementary biomarkers that could be measured by a standard ELISA workflow already accessible in routine laboratories. The primary objective of this study was to compare serum PSP levels across three groups: normotensive controls, GHT, and PE. The secondary objectives were to evaluate a comprehensive panel of systemic inflammatory indices among these groups, to assess the discriminatory performance of PSP in multiple clinically relevant scenarios by ROC analysis, and to determine whether PSP remains associated with PE within the HDP spectrum after adjustment for age, BMI, and gestational age. We hypothesized that serum PSP levels would be progressively elevated across the HDP spectrum, with significantly higher concentrations in PE compared to both GHT and normotensive pregnancies, and that this elevation would persist independently of conventional CBC-derived systemic inflammatory indices, providing discriminatory information not fully captured by routine inflammatory parameters.

## 2. Materials and Methods

### 2.1. Study Design and Setting

This prospective case–control study was conducted at the Department of Obstetrics and Gynecology, Ankara Etlik City Hospital, Ankara, Türkiye, between 5 March and 30 April 2026, following ethics committee approval granted on 3 March 2026 (Ethics Committee of Ankara Etlik City Hospital, Decision No: AEŞH-BADEK2-2026-084). Patient enrollment commenced two days after receipt of ethics approval. Serum samples were stored at −80 °C and batch-analyzed following completion of enrollment, and all procedures were conducted in compliance with the principles of the Declaration of Helsinki. Written informed consent was obtained from all participants. The study was designed and reported in accordance with the Strengthening the Reporting of Observational Studies in Epidemiology (STROBE) guidelines for case–control studies [22]; the completed STROBE checklist is provided as Supplementary Materials.

### 2.2. Participants

A total of 84 pregnant women were enrolled across three groups defined a priori. All participants were consecutive eligible women presenting to our department during the predefined enrollment period (5 March 2026 to 30 April 2026) who met the inclusion criteria and provided written informed consent. The PE group comprised 22 pregnant women diagnosed with PE according to ACOG 2020 diagnostic criteria [7] (systolic BP ≥140 mmHg or diastolic BP ≥90 mmHg on two occasions at least 4 h apart, combined with proteinuria ≥300 mg/24 h, or in the absence of proteinuria, evidence of new-onset thrombocytopenia, renal insufficiency, impaired liver function, pulmonary edema, or new-onset headache unresponsive to medication). Gestational age at diagnosis ranged from 33.5 to 38.8 weeks in the PE group; no lower gestational age limit was applied, as preterm PE represents a clinically significant disease phenotype.

The GHT group comprised 20 pregnant women with new-onset hypertension (systolic BP ≥140 mmHg or diastolic BP ≥90 mmHg on two occasions at least 4 h apart) after 20 weeks of gestation in the absence of proteinuria, end-organ dysfunction, or other features of severe disease, in accordance with ACOG and ISSHP criteria. The control group consisted of 42 healthy, normotensive women at term who were admitted for delivery. Because the control group comprised exclusively term pregnancies whereas the GHT and PE groups necessarily included earlier gestational ages reflecting the clinical course of these disorders, the study groups were not matched for gestational age; this mismatch is a recognized limitation of the design and is addressed explicitly when the PSP comparisons are interpreted (see Discussion and Limitations). All groups were between 18 and 40 years of age [7,8,23].

Exclusion criteria included: (1) pre-existing diabetes mellitus or pancreatitis; (2) chronic hypertension; (3) gestational age below 37 weeks (applicable to the control group only; preterm delivery in the PE and GHT groups was not an exclusion criterion, as it represents a recognized obstetric outcome of disease); (4) confirmed bacterial or viral infection within the preceding 2 weeks; (5) autoimmune disease; and (6) multiple gestation.

### 2.3. Sample Collection and PSP Measurement

Venous blood samples were collected at the time of clinical diagnosis, prior to any medical intervention in the PE and GHT groups (including, but not limited to, antihypertensive medication, magnesium sulfate, and antenatal corticosteroids), and prior to the onset of active labor in the control group, collected concurrently with routine admission blood sampling upon hospital presentation for delivery. This sampling protocol was designed to ensure that PSP and inflammatory index values reflected the underlying pathophysiological state of disease at presentation, uncontaminated by treatment-related effects. Following centrifugation at 3000 rpm for 10 min, using a Medwelt 800 D centrifuge (Medwelt, İzmir, Türkiye), serum was aliquoted and stored at −80 °C until analysis; storage duration ranged from approximately two weeks to two months, and all samples underwent a single freeze–thaw cycle prior to assay. Serum PSP concentrations were determined using a commercially available validated sandwich enzyme-linked immunosorbent assay (ELISA) kit specific for human PSP/REG1A (Bioassay Technology Laboratory, Jiaxing, Zhejiang, China; Cat. No. E6073Hu; standard curve range: 0.05–20 ng/mL; sensitivity: 0.029 ng/mL; intra-assay CV < 8%; inter-assay CV < 10%), performed in duplicate according to the manufacturer’s instructions, with the mean of duplicate readings used for analysis. Laboratory personnel performing the ELISAs were blinded to clinical group assignment. Recent studies have validated similar ELISA platforms for serum PSP measurement [24]. Intra-assay and inter-assay coefficients of variation were <8% and <10%, respectively.

### 2.4. Inflammatory Index Calculation

Complete blood count (CBC) parameters—absolute neutrophil count (N), lymphocyte count (L), monocyte count (Mo), and platelet count (Plt)—were obtained from venous samples collected simultaneously with serum samples and analyzed using an automated hematology analyzer (Sysmex XN-1000, Sysmex Corporation, Kobe, Japan). As specified in the exclusion criteria (Section 2.2), women with a confirmed bacterial or viral infection in the preceding two weeks were not enrolled, and all blood samples were drawn at the time of clinical diagnosis (GHT and PE) or on admission for delivery (controls) before the initiation of any antihypertensive therapy, magnesium sulfate, or delivery; the CBC-derived indices therefore reflect the underlying disease state rather than concurrent acute infection. The following indices were calculated using standard formulae as previously applied in obstetric and inflammatory research [12,13,25]:

**NLR = N/L | PLR = Plt/L | MLR = Mo/L | SII = (Plt × N)/L**

**SIRI = (Mo × N)/L | PIV = (Plt × N × Mo)/L | AISI = (N × Mo × Plt)/L^2^**

### 2.5. Power Analysis and Sample Size

Sample size was calculated using G*Power (v3.1) (Heinrich-Heine-Universität, Düsseldorf, Germany) for an independent two-sample t-test. Based on the mean PSP difference reported by Brun et al. (PE/HELLP mean 9.8 ng/mL versus healthy 7.9 ng/mL, pooled SD ~2.6 ng/mL) [21], yielding an estimated effect size of Cohen’s d ≈ 0.73 (medium-to-large), an alpha error of 0.05 and power of 0.80 produced an a priori required sample size of approximately 31 per group for the primary control versus PE comparison. Although our actual recruitment across the two-month enrollment period reflected the natural admission pattern of HDP cases at our tertiary center—with the control group exceeding the a priori target (*n* = 42) but the GHT (*n* = 20) and PE (*n* = 22) groups, whose enrollment was constrained by clinical incidence rather than recruitment design, falling somewhat below the per-group target of 31—the observed between-group effect sizes were substantially larger than the prior Brun-based estimate (Hedges’ g = 0.67 for control vs. GHT, indicating a medium effect; g = 1.41 for control vs. PE; g = 1.19 for GHT vs. PE, both indicating very large effects), which is consistent with a detectable PSP signal in the present cohort; however, these observed effect sizes should be interpreted descriptively, and the precision of PE-related estimates remains limited given the modest group sizes; these findings should therefore be considered exploratory, as the study was primarily powered for the control versus PE comparison and was not specifically powered for multivariable modeling or ROC cutoff validation. Hedges’ g (rather than Cohen’s d) was selected to provide an unbiased effect size estimate appropriate for small-to-moderate sample sizes. A sensitivity analysis indicated that the achieved sample sizes were sufficient to detect effect sizes of g ≥ 1.0 (very large) at α = 0.05 with 80% power for all PE-related comparisons; the smaller effect observed for control versus GHT is biologically consistent with the intermediate clinical phenotype of GHT within the HDP spectrum. The width of the bootstrap-derived confidence intervals around AUC estimates and odds ratios is reported in the relevant tables and should be considered when interpreting precision.

### 2.6. Statistical Analysis

Statistical analyses were performed using SPSS version 26.0 (IBM Corp., Armonk, NY, USA). Normality was assessed with the Shapiro–Wilk test. Three-group comparisons of normally distributed continuous variables were performed using one-way analysis of variance (ANOVA), expressed as mean ± SD; non-normally distributed variables were compared using the Kruskal–Wallis test, expressed as median (interquartile range, IQR). Significant overall results were followed by post hoc pairwise comparisons (independent-samples t-test or Mann–Whitney U test, as appropriate) with Bonferroni correction for multiple testing; reported pairwise *p* values are Bonferroni-adjusted. Categorical variables were compared with the chi-square or Fisher’s exact test. Associations between PSP and continuous clinical parameters were assessed using Spearman’s rank correlation coefficient (ρ), given the non-normal distribution of PSP. Receiver operating characteristic (ROC) curve analysis was performed across four clinical scenarios: PE versus control, PE versus GHT, PE versus all non-PE participants (control + GHT), and HDP (PE + GHT) versus control. NLR and SII were selected for direct ROC comparison with PSP in the primary PE-versus-control analysis as the two most widely cited CBC-derived inflammatory indices in the obstetric literature; ROC curves for the remaining indices were not generated as none reached statistical significance in the three-group comparisons, precluding meaningful discriminatory performance. AUC 95% confidence intervals were calculated using bootstrap resampling (2000 iterations). The area under the curve (AUC), sensitivity, specificity, positive predictive value (PPV), and optimal cutoff (Youden index) were reported. For each ROC analysis, the optimal cutoff was defined as the value maximizing the Youden index (J = sensitivity + specificity − 1). Differences in AUC between PSP and the two most widely cited reference indices (NLR and SII) in the primary PE-versus-control comparison were compared using the DeLong test for two correlated ROC curves. Binary logistic regression was performed to evaluate the association of PSP with PE (versus control + GHT combined) after adjustment for age, BMI, and gestational age, and multinomial logistic regression was performed with control as the reference category to model both GHT and PE simultaneously; given the limited number of PE (*n* = 22) and GHT (*n* = 20) cases, these regression analyses are presented as exploratory and hypothesis-generating. Model calibration was assessed using the Hosmer–Lemeshow goodness-of-fit test and model fit was quantified using the Nagelkerke R^2^ (binary) and McFadden Pseudo R^2^ (multinomial). Multicollinearity among the model covariates (PSP, maternal age, BMI, and gestational age) was assessed using variance inflation factors (VIF); all values were low (range 1.03–1.10, with the highest being that of PSP), well below the conventional threshold of 5, indicating no problematic collinearity among predictors. Overall fit of the multinomial model was additionally evaluated using the likelihood-ratio test against the intercept-only model. Internal validation of the binary logistic model was performed by bootstrap resampling (1000 replications), with optimism-corrected discrimination (area under the curve) reported. All tests were two-tailed, and statistical significance was set at *p* < 0.05. The reporting of diagnostic-accuracy components of the present analysis (ROC curves, sensitivity, specificity, predictive values, and discriminatory performance estimates) was guided by the principles of the STARD 2015 statement for studies of diagnostic accuracy.

## 3. Results

A total of 84 participants were enrolled across three groups: normotensive controls (*n* = 42), gestational hypertension (*n* = 20), and preeclampsia (*n* = 22); no exclusions were required during data collection, and complete laboratory and clinical data were available for all 84 participants (no missing values for any analyzed variable). The demographic and clinical characteristics of the three groups are presented in Table 1. The groups did not differ significantly in age (27.07 ± 4.78 vs. 28.16 ± 4.58 vs. 30.12 ± 5.46 years; ANOVA *p* = 0.068), height (*p* = 0.300), gravida (*p* = 0.728), or parity (*p* = 0.728). Body weight differed significantly across groups (73.6 ± 9.6 vs. 76.96 ± 8.9 vs. 82.92 ± 11.6 kg; *p* = 0.003), with corresponding BMI values showing a parallel stepwise gradient (28.01 ± 4.56 vs. 30.16 ± 4.20 vs. 31.50 ± 5.06 kg/m^2^; *p* = 0.015), reflecting the well-established association between elevated body weight and BMI with HDP risk. Gestational age at sampling decreased progressively across the HDP spectrum (39.17 ± 0.84 vs. 38.15 ± 1.14 vs. 36.80 ± 1.31 weeks; *p* < 0.001). Both systolic and diastolic blood pressures rose progressively from control to GHT to PE (*p* < 0.001 for both), confirming the categorical clinical separation defined by group inclusion criteria.

**Figure 1:**
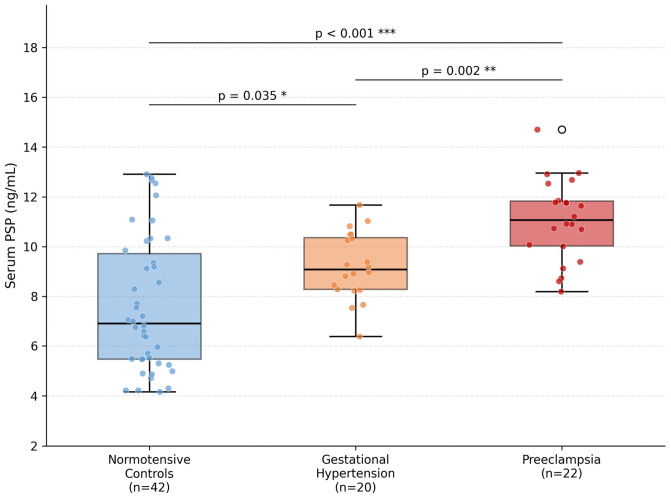
Box plots of serum PSP (ng/mL) across the three study groups: normotensive controls (*n* = 42), gestational hypertension (*n* = 20), and preeclampsia (*n* = 22). A stepwise elevation in PSP is observed across the spectrum of hypertensive disorders of pregnancy, with all three pairwise comparisons statistically significant after Bonferroni correction (Mann–Whitney U test). Boxes represent the interquartile range; horizontal lines represent the median; whiskers extend to 1.5× IQR; individual data points are overlaid. Statistical significance between groups is indicated as: * *p* < 0.05, ** *p* < 0.01, *** *p* < 0.001 (Mann–Whitney U test with Bonferroni correction).

The comparative analysis of serum PSP and systemic inflammatory indices across the three groups is summarized in Table 2 and visualized in Figure 1. Serum PSP showed a graded increase across the HDP spectrum: 6.91 (5.47–9.73) ng/mL in controls, 9.08 (8.29–10.36) ng/mL in GHT, and 11.07 (10.03–11.83) ng/mL in PE (Kruskal–Wallis H test, *p* < 0.001). All three pairwise post hoc comparisons (Mann–Whitney U test) remained statistically significant after Bonferroni correction: control vs. GHT (*p* = 0.035), control vs. PE (*p* < 0.001), and GHT vs. PE (*p* = 0.002). The control group median PSP value of 6.91 ng/mL is consistent with the reference range established for healthy pregnant women in the third trimester by Vonzun et al. [20]. A within-PE post hoc analysis showed no significant difference in PSP between preterm (<37 weeks; *n* = 9) and term (≥37 weeks; n = 13) cases [median 10.73 (9.39–10.92) vs. 11.77 (10.70–12.53) ng/mL; Mann–Whitney U, *p* = 0.109], suggesting that PSP elevation in PE is not primarily driven by gestational age at sampling.

In contrast to the robust between-group separation observed for PSP, none of the seven systemic inflammatory indices reached statistical significance across the three groups (all *p* > 0.05; Table 2). However, several indices exhibited a numeric upward trend consistent with the disease-severity gradient (control < GHT < PE). MLR rose progressively from 0.27 (0.15–0.40) in controls to 0.30 (0.19–0.59) in GHT and 0.35 (0.22–0.46) in PE (*p* = 0.072), and SIRI showed a similar pattern (1.54 [1.02–2.64] vs. 2.30 [1.04–4.56] vs. 2.32 [1.46–3.79]; *p* = 0.071), with both indices approaching but not reaching the conventional significance threshold. NLR was higher in both hypertensive groups compared with controls (3.21 [2.13–4.70] vs. 4.66 [1.91–7.28] vs. 4.04 [2.88–6.51]; *p* = 0.192), reflecting a trend toward relative lymphopenia and neutrophilia in HDP. In contrast, platelet-containing composite indices behaved differently: PLR (133.10 vs. 155.58 vs. 120.81; *p* = 0.399) and SII (766.6 vs. 1138.9 vs. 801.1; *p* = 0.603) showed a non-monotonic pattern with relative attenuation in the PE group, plausibly reflecting the modest thrombocytopenia observed in established PE that depresses ratios incorporating platelet count. PIV (383.8 vs. 542.8 vs. 444.2; *p* = 0.266) and AISI (271.1 vs. 280.4 vs. 258.5; *p* = 0.232) showed similar non-monotonic patterns. Taken together, these findings indicate that PSP appears to capture a graded biological signal that was not captured by the conventional CBC-derived inflammatory indices, which showed numerically lower discriminatory performance at the time of clinical PE diagnosis in this cohort.

Absolute hemogram parameters are compared in Table 3. None of the leukocyte subpopulations reached statistical significance across the three groups (all *p* > 0.05); however, the PE group exhibited the highest WBC (10.20 ± 2.87) and neutrophil counts (7.52 ± 2.64), together with the lowest lymphocyte counts (1.72 ± 0.69), reflecting a mild stress-related leukocytic response with a relative lymphopenic shift compared with normotensive controls. Platelet count was modestly lower in the PE group (210.5 ± 49.8) compared with controls (253.9 ± 75.9) and GHT (256.3 ± 62.2) (ANOVA *p* = 0.033), consistent with the mild thrombocytopenia component of PE pathophysiology described in recent platelet-focused analyses [26]. The numeric trends observed in raw hemogram parameters (mild leukocytosis and relative lymphopenia in PE) are consistent with the parallel borderline trends in derived inflammatory indices (Table 2), suggesting a low-grade systemic inflammatory shift in established HDP that does not, however, reach the statistical threshold required for reliable clinical discrimination.

**Figure 2:**
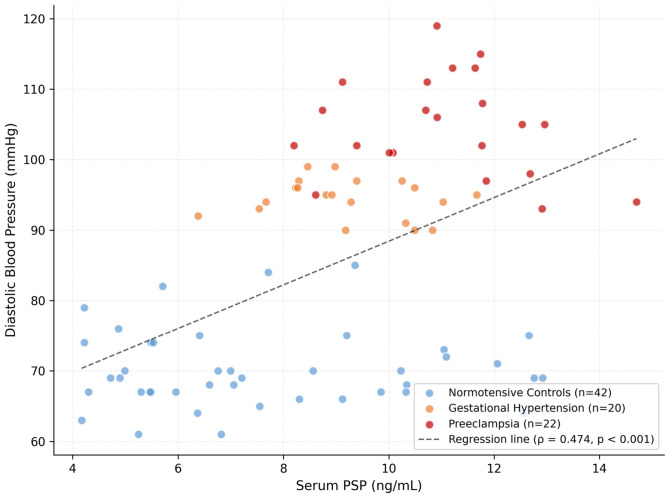
Scatter plot illustrating the relationship between serum PSP levels and diastolic blood pressure across the full study cohort (*n* = 84). Blue: normotensive controls; orange: gestational hypertension; red: preeclampsia. The dashed regression line reflects the full-cohort Spearman correlation (*ρ* = 0.474; *p* < 0.001), driven by categorical group separation across the HDP spectrum.

Spearman rank correlation analysis between serum PSP and both clinical parameters and systemic inflammatory indices is presented in Table 4. As illustrated in Figure 2, positive full-cohort correlations emerged between PSP and systolic blood pressure (ρ = 0.423; *p* < 0.001) and between PSP and diastolic blood pressure (ρ = 0.474; *p* < 0.001), alongside a modest negative correlation with gestational age (ρ = −0.266; *p* = 0.015) reflecting earlier delivery in the PE group. None of these associations reached significance within any individual diagnostic group, indicating that the full-cohort correlations primarily reflect categorical between-group separation across the HDP spectrum rather than continuous within-group relationships. Importantly, PSP showed no significant correlation with any of the seven systemic inflammatory indices in either the full cohort or the within-PE subgroup (all |ρ| ≤ 0.20; all *p* > 0.05), and the small absolute magnitudes (the largest was NLR ρ = +0.190 within the PE subgroup) indicate that the modest inflammatory trends observed at the group level are not paralleled by a meaningful within-patient linear relationship between PSP- and CBC-derived markers. This dissociation at the individual-patient level supports the interpretation that PSP captures a pathophysiological signal that is complementary to the inflammatory information conveyed by conventional leukocyte–platelet indices and, in this cohort, was not paralleled by significant differences in those indices.

**Figure 3:**
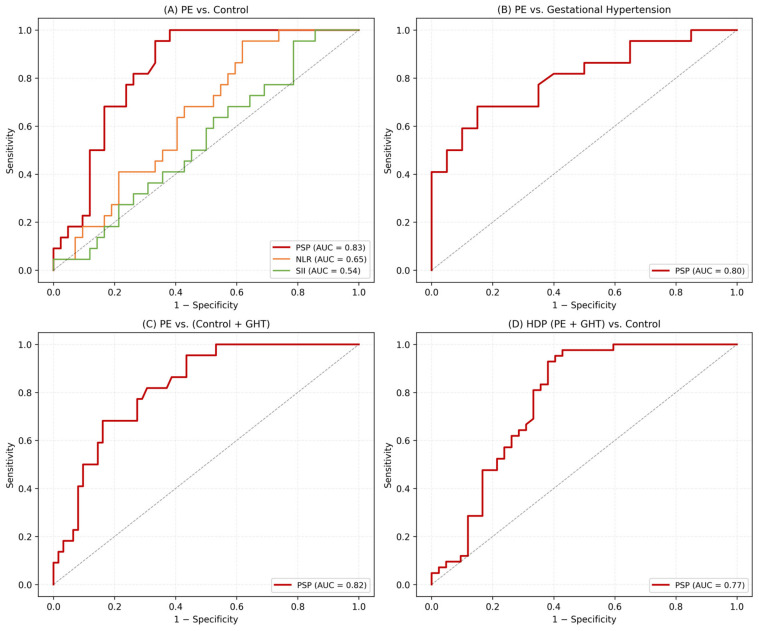
Receiver operating characteristic (ROC) curves of PSP across four clinical scenarios. (**A**) PE versus normotensive control, with NLR and SII shown for comparison; (**B**) PE versus gestational hypertension; (**C**) PE versus all non-PE participants (control + GHT); (**D**) HDP (PE + GHT) versus control. PSP demonstrates moderate-to-good discriminatory performance across all scenarios, while inflammatory indices (NLR, SII) achieve substantially lower discriminatory performance even in the simplest PE-versus-control comparison. In each panel, the diagonal dotted line indicates the reference line of no discrimination (AUC = 0.50).

ROC analysis was performed across four clinical scenarios to characterize the discriminatory performance of PSP, with NLR and SII included for comparison in the primary PE-versus-control analysis (Table 5; Figure 3). For PE versus normotensive controls, PSP yielded an AUC of 0.83 (95% CI: 0.73–0.92; *p* < 0.001), with 95.5% sensitivity and 66.7% specificity at the optimal Youden cutoff of 8.61 ng/mL, corresponding to a high negative predictive value (NPV 96.6%) but a modest positive predictive value (PPV 48.8%) within this comparison. NLR yielded a modest AUC of 0.65 (95% CI: 0.51–0.78) and SII performed at chance level (AUC 0.54; 95% CI: 0.40–0.69), both substantially below PSP and neither attaining statistical significance after Bonferroni adjustment. The superiority of PSP over each index in this comparison was confirmed by the DeLong test for correlated ROC curves (PSP vs. NLR, *p* = 0.023; PSP vs. SII, *p* = 0.001), and this advantage remained statistically significant in the PE-versus-GHT and PE-versus-non-PE comparisons. For the clinically critical task of differentiating PE from GHT, PSP achieved an AUC of 0.80 (95% CI: 0.66–0.93; *p* = 0.001), with 68.2% sensitivity and 85.0% specificity at a cutoff of 10.70 ng/mL (PPV 82.4%, NPV 70.8%). For PE versus all non-PE participants (control + GHT combined), PSP yielded an AUC of 0.82 (95% CI: 0.73–0.91; *p* < 0.001), and for HDP (PE + GHT) versus control, PSP achieved an AUC of 0.77 (95% CI: 0.67–0.86; *p* < 0.001). Predictive values for all scenarios are summarized in Table 5.

Binary logistic regression with PE as the outcome (versus control + GHT combined) showed that serum PSP remained associated with PE after adjustment for age, BMI, and gestational age (Table 6). Each 1 ng/mL increase in PSP was associated with a 132% increase in the odds of PE (OR 2.32; 95% CI: 1.29–4.16; *p* = 0.005). Gestational age was inversely associated with PE (OR 0.21; 95% CI: 0.08–0.51; *p* = 0.001), while age and BMI were not independently associated with PE in the adjusted model. Notably, the absence of an independent BMI effect indicates that the elevated PSP in PE is not explained by group differences in maternal adiposity. The model demonstrated excellent calibration (Hosmer-Lemeshow χ^2^ = 4.18, df = 8, *p* = 0.840) and good discrimination (Nagelkerke R^2^ = 0.726). The events-per-variable ratio for this model was modest (approximately 5.5:1); nonetheless, bootstrap internal validation (1000 replications) indicated minimal optimism (0.01), with an optimism-corrected AUC of 0.94 against an apparent AUC of 0.96, suggesting that the model’s discrimination was not materially inflated by overfitting. Given the modest number of PE and GHT cases, these regression analyses should be interpreted as exploratory and hypothesis-generating.

Multinomial logistic regression with control as the reference category (Table 7) showed a consistent graded association between PSP and HDP severity, although these findings should be interpreted cautiously given the modest sample size and potential model instability related to limited event counts. Each 1 ng/mL increase in PSP was associated with a 42% increase in the odds of GHT versus control (OR 1.42; 95% CI: 1.05–1.92; *p* = 0.022) and a 199% increase in the odds of PE versus control (OR 2.99; 95% CI: 1.57–5.68; *p* = 0.001). Gestational age remained inversely associated with both conditions, with a substantially stronger effect in PE (OR 0.08 vs. 0.29 for GHT). Age and BMI were not independently associated with either condition, suggesting that the graded association between PSP and HDP severity is not explained by maternal adiposity. The model achieved a McFadden Pseudo R^2^ of 0.470 with overall LLR *p* < 0.001, indicating improved model fit compared to the null model. Given the modest event counts in the GHT and PE groups, these multinomial estimates should be interpreted as exploratory.

## 4. Discussion

In this prospective case–control study of 84 pregnant women across the spectrum of hypertensive disorders of pregnancy, we report three principal findings. First, serum PSP exhibited a graded, stepwise elevation across the three groups (control < GHT < PE), with all three pairwise comparisons statistically significant after Bonferroni correction. Second, PSP achieved moderate-to-good discriminatory performance not only for PE versus normotensive controls (AUC 0.83) but, critically, for the differential diagnosis of PE versus GHT (AUC 0.80)—a clinically important distinction within the HDP spectrum. Third, none of the seven contemporary CBC-derived systemic inflammatory indices (NLR, PLR, MLR, SII, SIRI, PIV, AISI) reached statistical significance, although several (notably MLR and SIRI) demonstrated borderline upward trends consistent with the disease-severity gradient. PSP therefore appears to capture a graded biological signal that was not paralleled by significant differences in the CBC-derived inflammatory indices, which showed numerically lower discriminatory performance in this cohort (NLR AUC 0.65; SII AUC 0.54). Importantly, the non-significance of these indices should not be read as evidence of their inferiority; with only 20 GHT and 22 PE participants the study was underpowered to detect the modest index differences typically reported for these markers, and the AUC advantage of PSP over NLR and SII was statistically significant on DeLong testing (*p* = 0.023 and *p* = 0.001, respectively, in the PE-versus-control comparison); this finding nonetheless derives from a modestly sized cohort and requires confirmation, so that PSP is best positioned as a discriminating marker that complements, rather than replaces, established assessment. This pattern indicates that PSP may reflect subclinical inflammatory and stress-related processes that are only weakly captured by CBC-derived markers. The relatively modest positive predictive value (48.8%) observed in the PE-versus-control comparison likely reflects the relatively low prevalence of preeclampsia within the study cohort and underscores the importance of validating PSP performance across populations with varying disease prevalence before clinical implementation. These findings should be interpreted in the context of discrimination rather than calibration performance, and further studies incorporating calibration metrics and decision-curve analysis are warranted to define clinical utility.

PSP across the HDP spectrum: a graded association. The observed gradient in PSP levels (6.91 → 9.08 → 11.07 ng/mL) is consistent with a graduated pathophysiological response that parallels disease severity within the HDP continuum. This pattern was further reflected in the multinomial logistic regression, in which each 1 ng/mL increase in PSP raised the odds of GHT by 42% and the odds of PE by 199%, both versus normotensive controls. The roughly two-fold higher OR for PE compared with GHT mirrors the categorical clinical severity gradient and is consistent with the conceptualization of PSP as a quantitative biomarker that may track disease intensity, rather than binary disease status alone. Such graded behavior is consistent with biologically meaningful biomarkers and supports further investigation of PSP as a candidate marker that may reflect the underlying pathophysiological burden across the spectrum of pregnancy-associated hypertension. An important consideration when interpreting this gradient is that the normotensive control group consisted exclusively of women at term, whereas the GHT and PE groups included earlier gestational ages reflecting the natural clinical course of these conditions; this gestational-age mismatch represents a potential source of bias even though our within-PE post hoc comparison showed no significant PSP difference between preterm and term cases (*p* = 0.109), and even though the magnitude of physiological PSP variation across trimesters reported by Vonzun et al. [20] (~1.4 ng/mL) is markedly smaller than the between-group differences observed in our cohort (PE vs. controls: ~4.2 ng/mL). A further consideration is that BMI also differed significantly across the three groups (28.0 vs. 30.2 vs. 31.5 kg/m^2^; *p* = 0.015), which is biologically expected given the well-established role of obesity as both an independent risk factor for PE and a contributor to chronic low-grade inflammation; however, BMI did not retain independent predictive value in either the binary or multinomial regression models, indicating that the elevated PSP observed in PE is not adequately explained by group differences in maternal adiposity, and arguing that the PSP gradient across the HDP spectrum reflects HDP-specific pathophysiology rather than a generic obesity-driven inflammatory response. Given the modest sample size, these regression-based estimates should nevertheless be interpreted as exploratory.

The potential influence of gestational age on PSP concentrations warrants specific consideration, and three complementary lines of evidence argue against gestational-age mismatch as an explanation for the observed gradient. First, the magnitude of any gestational-age effect is small relative to the between-group differences we observed: the trimester-specific reference values reported by Vonzun et al. [20] (first trimester 6.96 ng/mL to third trimester 8.34 ng/mL) correspond to an average change of only approximately 0.05–0.06 ng/mL per gestational week, so that the ~2.4-week difference in mean gestational age between our control (39.2 weeks) and PE (36.8 weeks) groups would predict a gestational-age-attributable PSP difference of only ~0.14 ng/mL—less than 4% of the ~4.2 ng/mL difference actually observed between these groups; because this estimate derives from the average slope across the whole of pregnancy rather than the flatter changes typically seen near term, it most likely over-states the true near-term contribution. Second, and importantly, the direction of the gestational-age mismatch is conservative rather than favorable to our hypothesis: because PSP rises with advancing gestation, gestational-age-matched controls sampled at the earlier gestations characteristic of the PE group would be expected to show equal or lower PSP than our term controls, which would widen rather than narrow the observed gap; the elevation seen in the earlier-gestation PE group therefore occurs against, not with, the expected direction of any gestational-age effect. Third, gestational age was entered as a covariate in both the binary and multinomial regression models, in which PSP retained a strong and independent association with PE (OR 2.32 and 2.99, respectively) while gestational age itself remained independently associated—indicating that the two effects are statistically separable and that the PSP–PE association does not simply reflect gestational age acting through PSP. Taken together, these considerations make it unlikely that the observed PSP gradient is an artifact of gestational-age differences; nonetheless, as detailed in the limitations, the absence of prospectively enrolled gestational-age-matched normotensive controls remains a genuine constraint on causal interpretation, and validation in a design that samples controls at the same gestational ages as cases is a priority for future work.

PSP discriminates PE from GHT—a clinically essential distinction. Differentiating PE from GHT remains a persistent diagnostic challenge in clinical practice. Both conditions present with new-onset hypertension after 20 weeks of gestation, yet they differ substantially in maternal–fetal risk, surveillance intensity, delivery timing, and long-term cardiovascular sequelae. To our knowledge, this is among the first studies to evaluate PSP specifically for the differential discrimination of PE from GHT within the HDP spectrum. With an AUC of 0.80 (95% CI: 0.66–0.93) and a specificity of 85.0% at a cutoff of 10.70 ng/mL, PSP demonstrated potentially clinically relevant discriminatory performance for PE versus GHT. From a triage perspective, a PSP value at or above this exploratory threshold in a pregnant woman presenting with new-onset hypertension after 20 weeks could—pending external validation, and only within future validated pathways—flag the patient for closer maternal–fetal surveillance, expedited workup, and lower thresholds for hospital admission, whereas values clearly below this range might conceptually support outpatient monitoring of suspected GHT phenotypes; such risk-tier triage could plausibly shorten time to PE recognition in settings where angiogenic testing is unavailable. This finding has direct relevance to risk stratification: in the absence of universally available angiogenic testing (sFlt-1/PlGF)—a marker reported to be elevated in PE but not in GHT, supporting fundamental pathophysiological differences between the two conditions [27]—a single serum measurement at the time of clinical presentation could provide objective, biomarker-based evidence to inform whether observed hypertension represents the milder GHT phenotype or evolving PE. The supporting multinomial regression results, in which each 1 ng/mL rise in PSP was associated with a 42% higher odds of GHT and an approximately threefold higher odds of PE versus normotensive controls, should, however, be interpreted with particular caution. The modest event counts in the GHT (*n* = 20) and PE (*n* = 22) groups increase the potential for model instability in multivariable analyses, placing these multinomial estimates firmly within an exploratory, hypothesis-generating framework rather than supporting definitive risk-stratification thresholds. Further validation in larger multicenter cohorts is essential before clinical adoption can be considered.

The mechanistic basis for PSP elevation in PE merits detailed consideration. The mechanisms outlined in this paragraph are hypothetical, are not tested by the present clinical study, and are presented solely to frame directions for future work. PSP (REG1A) is structurally classified as a C-type lectin-like protein, a family of calcium-dependent glycan-binding proteins critically involved in innate immunity, leukocyte trafficking, and cellular stress responses [16,17]. PSP has been reported to activate polymorphonuclear neutrophils (PMNs) via receptor-mediated binding, modulate the NLRP3 inflammasome, and regulate macrophage function during sterile and infectious inflammation [17,28]. Within the context of PE, several non-mutually exclusive mechanisms could account for the observed PSP elevation. First, placental ischemia–reperfusion injury characteristic of PE generates oxidative stress and damage-associated molecular patterns that may signal to the pancreas via systemic inflammatory cytokines, prompting acinar and beta-cell PSP secretion. Mechanistically, hypoxia-driven release of soluble mediators such as TNF-α, IL-6, and HMGB1 from the ischemic placenta could plausibly reach the pancreatic microenvironment through the maternal circulation and engage TLR-4 and inflammasome-dependent pathways within acinar cells, thereby upregulating Reg3-family genes including PSP/Reg1A. Such a model would frame PSP elevation in PE not as a pancreas-specific event but rather as a downstream readout of placenta-driven sterile inflammatory signaling distributed across the maternal compartment. Second, given that the NLRP3 inflammasome is implicated in PE pathophysiology, PSP may reflect, rather than directly mediate, trophoblast-derived sterile inflammatory signaling within this pathway. Third, the placenta–pancreas axis may operate through direct hormonal signaling: trophoblast-derived factors including syncytiotrophoblast extracellular vesicles can influence pancreatic islet function during pregnancy, providing a plausible inter-organ pathway for PSP induction. Convergent support for the existence of such a pregnancy-specific placenta-pancreas signaling axis comes from a recent prospective cohort study from our institution, conducted in an entirely separate, non-overlapping cohort and time period, which demonstrated that serum PSP is also elevated in gestational diabetes mellitus relative to normoglycaemic pregnancy [29]; the GDM context—characterized by β-cell endoplasmic reticulum stress in response to placental hormone-driven insulin resistance—and the PE context investigated here may therefore represent two distinct clinical manifestations of pregnancy-related stress signaling reaching the pancreatic compartment, even though the underlying triggers (metabolic vs. ischemic–inflammatory) differ. These mechanistic interpretations remain speculative and should be regarded as hypothesis-generating, as the present clinical study did not directly assess placental, pancreatic, or inflammasome-related pathways. Experimental and translational studies—including in vitro trophoblast-pancreatic co-culture models and animal models of placental ischemia—are required to clarify whether PSP elevation reflects placenta–pancreas inter-organ signaling, sterile inflammatory activation, or a broader systemic stress response in preeclampsia [17,19].

The contrast between PSP and conventional CBC-derived inflammatory indices in our cohort is informative. Whereas PSP reliably distinguished PE and GHT from normotensive pregnancy, the seven inflammatory indices captured the same disease-severity gradient only as borderline numeric trends (MLR *p* = 0.072; SIRI *p* = 0.071). The modest thrombocytopenia present in established PE additionally suppressed platelet-containing composite indices (PLR and SII), producing non-monotonic patterns that further blurred their discriminatory value. Taken together, PSP appears to capture a broader pregnancy-related stress or placenta–organ signaling response that is not adequately represented by conventional CBC-derived inflammatory indices. These findings are consistent with the heterogeneity reported in the literature: while some first-trimester studies have documented predictive value for SIRI, PIV, or SII [12,13], multiple peripartum studies have reported only modest or non-significant index differences in established PE compared to controls—consistent with the borderline trends observed in our cohort [15]. The pattern observed in our cohort suggests that, at the time of clinical PE diagnosis, the maternal systemic inflammatory milieu shows a directional but quantitatively muted shift compared with classical infectious or sepsis-related inflammation, and that biomarkers reflecting placenta–organ signaling—such as PSP—may capture and amplify aspects of PE biology that lie beyond what CBC-derived indices can register. A complementary explanation is that systemic inflammatory indices may be most informative when measured in the first or early second trimester—well before the clinical onset of PE—when subclinical immunological priming and trophoblast-related inflammation are evolving. By the peripartum stage, when our samples were obtained, these indices may have returned toward population norms or may be obscured by physiological pregnancy-related leukocytosis. This temporal pattern could reconcile the discrepancy between earlier predictive studies and our peripartum negative findings, while also highlighting that PSP elevation is detectable at the time of established disease—a clinically useful diagnostic window.

In comparison with the established angiogenic markers, the proposed clinical utility of PSP is complementary rather than competitive. PSP should therefore be considered a complementary rather than a replacement biomarker within existing diagnostic frameworks. Without direct comparison against angiogenic biomarkers, particularly the sFlt-1/PlGF ratio, PSP cannot yet be positioned within current PE decision algorithms. The sFlt-1/PlGF ratio remains the most extensively validated biomarker for PE risk stratification and short-term prediction [30], yet its widespread implementation is constrained by assay availability and cost in many settings. PSP, measurable by ELISA at low cost and amenable to existing immunoassay platforms [31], may serve as a practical adjunctive marker in resource-limited settings or as a complementary tool in settings where angiogenic testing is available. The performance of PSP in differentiating PE from GHT (AUC 0.80) compares favorably with other proposed differential markers and warrants direct head-to-head comparison with sFlt-1/PlGF in future multicenter studies.

This study has several limitations that should be acknowledged. First, the relatively modest sample sizes of the GHT (*n* = 20) and PE (*n* = 22) groups, dictated by recruitment within a single two-month enrollment window at a single center, limit the precision of effect estimates and may reduce the generalizability of our findings. Given the limited number of PE and GHT cases, the multivariable binary and multinomial regression analyses presented here should be interpreted as exploratory and hypothesis-generating; the stability of regression estimates and ROC-derived thresholds is constrained by the modest event count, and the proposed PSP cutoffs should not be considered clinically actionable until externally validated. The defined two-month recruitment window at a single tertiary center may also introduce selection bias related to seasonal, institutional, or specific admission-pattern variability common to time-defined single-center recruitment. Consequently, the findings may not fully represent the broader HDP population, and the specific PSP cutoff values derived from this cohort might not be directly generalizable to other geographic regions or populations with different demographic characteristics and baseline disease prevalences. Second, although gestational age differed significantly across groups, this pattern is partly inherent to the clinical course of PE and GHT. Our within-PE post hoc analysis demonstrated no significant difference in PSP between preterm and term PE cases (*p* = 0.109), arguing against gestational age as the sole driver of PSP elevation. Nevertheless, residual confounding by gestational age cannot be fully excluded, particularly given the modest subgroup sizes and the fact that the control group consisted only of normotensive women at term, so that gestational-age-matched normotensive controls—particularly for earlier gestational ages corresponding to preterm PE and GHT cases—were not available; this represents an important limitation, especially because PSP levels may vary modestly across pregnancy. The natural increase in PSP across trimesters reported by Vonzun et al. (first trimester 6.96 ± 2.5 ng/mL, second 7.43 ± 2.21 ng/mL, third 8.34 ± 2.69 ng/mL) [20] suggests that gestational-age-related variation alone (~1.4 ng/mL across trimesters) is markedly smaller in magnitude than the between-group differences observed in our cohort (PE vs. controls: ~4.2 ng/mL), arguing against gestational age as a primary explanation for the observed PSP gradient. Third, blood samples were collected at the time of hospital admission, in many cases close to the onset of active labor; while this reflects the real-world clinical context in which PE is most commonly diagnosed and biomarker testing would be applied, it does not allow distinction between early- and late-onset disease windows. Fourth, we did not measure angiogenic biomarkers (sFlt-1, PlGF, or the sFlt-1/PlGF ratio), precluding direct comparison with the current reference biomarker framework and preventing assessment of whether PSP adds incremental value beyond established angiogenic testing; in particular, no head-to-head AUC comparison or reclassification analysis (for example, net reclassification improvement) against the sFlt-1/PlGF ratio was possible, so any incremental advantage of PSP over the established angiogenic framework remains to be demonstrated. Fifth, the absence of repeated longitudinal sampling prevents inference about trajectory changes in PSP through gestation. Sixth, maternal and perinatal outcome data (mode of delivery, birth weight, Apgar scores, neonatal intensive-care admission, and composite adverse outcomes) were not collected, so the prognostic value of PSP and its relationship to disease severity could not be assessed. Seventh, the cohort was not stratified by onset timing (early- versus late-onset PE) or by disease severity (mild versus severe features per ACOG criteria), which limits mechanistic and subgroup-specific inference. Despite these limitations, this study should be regarded as a hypothesis-generating proof-of-concept investigation that establishes the differential discriminatory capacity of PSP within the HDP spectrum. The very large effect sizes observed for the primary PE-related comparisons (Hedges’ g = 1.19–1.41) and the consistent graded gradient across both univariable and multivariable analyses provide a foundation for subsequent large-scale, multicenter validation studies. The proposed PSP thresholds (8.61 and 10.70 ng/mL) require external validation in independent populations and should not be interpreted as clinically actionable cutoffs at this stage. Future research should address these limitations through larger multicenter cohorts, gestational-age-matched normotensive controls, longitudinal sampling across trimesters, head-to-head comparison with angiogenic markers, and external validation of the proposed cutoffs in independent populations.

From a clinical-translational standpoint, the immediate next steps include external validation of the PSP cutoffs proposed here, evaluation of PSP performance in earlier gestational windows (potentially as a screening tool), and direct comparison with sFlt-1/PlGF in mixed cohorts. Filippidis et al. recently demonstrated the value of serial PSP monitoring in critical illness [32]; an analogous strategy of serial measurement during pregnancy may capture the trajectory of pathophysiological evolution and improve early identification of women progressing from GHT to PE. Future studies integrating PSP with angiogenic and endothelial biomarkers may help define multi-marker models for HDP stratification.

## 5. Conclusions

Serum pancreatic stone protein exhibits a graded elevation across the spectrum of hypertensive disorders of pregnancy, with stepwise increases from normotensive controls to gestational hypertension to preeclampsia. Beyond differentiating PE from normotensive pregnancy, PSP showed exploratory discrimination of PE from GHT (AUC 0.80), representing, to our knowledge, among the first focused evaluations of PSP for this differential diagnostic task. Although several conventional CBC-derived inflammatory indices (NLR, PLR, MLR, SII, SIRI, PIV, AISI) showed borderline numeric trends across the HDP spectrum, none reached statistical significance, indicating that PSP captures a discriminatory signal that reflected discriminatory information not captured by the CBC-derived markers, which showed numerically lower discriminatory performance in this cohort. This more consistent and statistically robust gradient, combined with the graded association across the HDP spectrum, represents one of the most distinctive features of PSP as a candidate HDP biomarker and supports the view that PSP may offer additional discriminatory information beyond that provided by routinely available CBC-derived inflammatory indices, pending validation in larger cohorts. The proposed PSP thresholds (8.61 and 10.70 ng/mL) should be regarded strictly as exploratory. Clinicians should not adopt these values for clinical decision-making or patient triage until they have been independently validated in prospective, multicenter studies across diverse populations. Future research priorities should specifically include prospective validation of these thresholds in independent populations of differing ethnic, geographic, and risk-profile composition, evaluation across populations with varying disease prevalence to refine positive and negative predictive value estimates, head-to-head benchmarking against the established sFlt-1/PlGF ratio, and longitudinal assessment of PSP trajectories from early pregnancy through delivery in cohorts with gestational-age-matched normotensive controls. While these findings require confirmation in larger multicenter prospective studies, they support further investigation of PSP as a candidate biomarker for the differential assessment and risk stratification of hypertensive disorders of pregnancy.

## Figures and Tables

**Table 1:** Demographic and clinical characteristics of study groups.

Variable	Control (*n* = 42)	GHT (*n* = 20)	Preeclampsia (*n* = 22)	*p* Value	Test
Age (years), mean ± SD	27.07 ± 4.78	28.16 ± 4.58	30.12 ± 5.46	0.068	ANOVA
Gestational age (weeks)	39.17 ± 0.84	38.15 ± 1.14	36.80 ± 1.31	<0.001 *	ANOVA
Height (cm)	162.5 ± 6.3	160.1 ± 6.0	162.6 ± 5.9	0.300	ANOVA
Weight (kg)	73.6 ± 9.6	76.96 ± 8.9	82.92 ± 11.6	0.003 *	ANOVA
BMI (kg/m^2^)	28.01 ± 4.56	30.16 ± 4.20	31.50 ± 5.06	0.015 *	ANOVA
Gravida, median (IQR)	2 (1–3)	2 (1–2)	2 (1–2)	0.728	K-W
Parity, median (IQR)	1 (0–2)	1 (0–1)	1 (0–1)	0.728	K-W
Systolic BP (mmHg)	114 (107–119)	150 (146–153)	167 (164–173)	<0.001 *	K-W
Diastolic BP (mmHg)	69 (67–74)	95 (93–96)	105 (101–110)	<0.001 *	K-W

Normally distributed continuous variables were compared using one-way ANOVA; non-normally distributed variables were compared using the Kruskal–Wallis test (K-W). Abbreviations: SD: standard deviation; IQR: interquartile range; GHT: gestational hypertension; BP: blood pressure. * *p* < 0.05 is considered statistically significant.

**Table 2:** Serum PSP and systemic inflammatory index comparison among study groups.

Parameter	Control (*n* = 42)	GHT (*n* = 20)	Preeclampsia (*n* = 22)	*p* Value	Test
PSP (ng/mL)	6.91 (5.47–9.73)	9.08 (8.29–10.36)	11.07 (10.03–11.83)	<0.001 *	K-W
NLR	3.21 (2.13–4.70)	4.66 (1.91–7.28)	4.04 (2.88–6.51)	0.192	K-W
PLR	133.10 (85.46–186.67)	155.58 (99.89–255.32)	120.81 (73.55–201.86)	0.399	K-W
MLR	0.27 (0.15–0.40)	0.30 (0.19–0.59)	0.35 (0.22–0.46)	0.072	K-W
SII	766.6 (550.3–1330.6)	1138.9 (529.2–1652.7)	801.1 (575.1–1363.0)	0.603	K-W
SIRI	1.54 (1.02–2.64)	2.30 (1.04–4.56)	2.32 (1.46–3.79)	0.071	K-W
PIV	383.8 (169.3–722.6)	542.8 (229.9–1026.8)	444.2 (294.8–861.0)	0.266	K-W
AISI	271.1 (66.8–356.7)	280.4 (79.2–809.5)	258.5 (152.5–619.4)	0.232	K-W

Values are median (IQR). Abbreviations: PSP: pancreatic stone protein; NLR: neutrophil-to-lymphocyte ratio; PLR: platelet-to-lymphocyte ratio; MLR: monocyte-to-lymphocyte ratio; SII: systemic immune-inflammation index; SIRI: systemic inflammation response index; PIV: pan-immune inflammation value; AISI: aggregate index of systemic inflammation; GHT: gestational hypertension; K-W: Kruskal–Wallis test. * *p* < 0.05 is considered statistically significant.

**Table 3:** Comparison of hemogram parameters among study groups.

Parameter	Control (*n* = 42)	GHT (*n* = 20)	Preeclampsia (*n* = 22)	*p* Value	Test
WBC (10^3^/µL)	9.56 ± 2.79	9.47 ± 2.25	10.20 ± 2.87	0.607	ANOVA
Neutrophil (10^3^/µL)	6.77 ± 2.72	6.75 ± 2.26	7.52 ± 2.64	0.508	ANOVA
Lymphocyte (10^3^/µL)	2.05 ± 0.79	1.81 ± 0.82	1.72 ± 0.69	0.219	ANOVA
Monocyte (10^3^/µL)	0.53 ± 0.30	0.55 ± 0.16	0.58 ± 0.18	0.718	ANOVA
Platelet (10^3^/µL)	253.9 ± 75.9	256.3 ± 62.2	210.5 ± 49.8	0.033 *	ANOVA

Abbreviations: WBC: white blood cell count; GHT: gestational hypertension. * *p* < 0.05 is considered statistically significant.

**Table 4:** Spearman rank correlation coefficients between serum PSP and clinical parameters/systemic inflammatory indices.

Variable	Full Cohort ρ	*p*	Within-PE ρ	*p*
**Clinical parameters**				
Systolic BP (mmHg)	+0.423	<0.001 *	−0.143	0.526
Diastolic BP (mmHg)	+0.474	<0.001 *	−0.189	0.399
Gestational age (weeks)	−0.266	0.015 *	+0.245	0.272
BMI (kg/m^2^)	+0.201	0.067	+0.130	0.563
Age (years)	+0.098	0.374	+0.128	0.570
**Systemic inflammatory indices**				
NLR	+0.112	0.312	+0.190	0.396
PLR	−0.061	0.581	−0.047	0.836
MLR	+0.110	0.320	−0.163	0.468
SII	−0.027	0.810	+0.135	0.549
SIRI	+0.111	0.314	−0.007	0.974
PIV	+0.013	0.906	−0.041	0.856
AISI	+0.058	0.602	−0.005	0.982

Abbreviations: ρ: Spearman rank correlation coefficient; PE: preeclampsia; BP: blood pressure; BMI: body mass index; NLR: neutrophil-to-lymphocyte ratio; PLR: platelet-to-lymphocyte ratio; MLR: monocyte-to-lymphocyte ratio; SII: systemic immune-inflammation index; SIRI: systemic inflammation response index; PIV: pan-immune inflammation value; AISI: aggregate index of systemic inflammation. Full cohort: *n* = 84; Within-PE: *n* = 22. Within-control and within-GHT correlations were also non-significant for all variables. Note the absence of any significant correlation between PSP and the seven inflammatory indices, supporting the dissociation of PSP from generalized leukocyte–platelet inflammation. * *p* < 0.05 is considered statistically significant.

**Table 5:** Diagnostic performance of PSP and inflammatory indices: ROC analysis across clinical scenarios.

Comparison	Biomarker	AUC (95% CI)	Sens. (%)	Spec. (%)	Cutoff	PPV (%)	NPV (%)	*p* Value
PE vs. Control	PSP	0.83 (0.73–0.92)	95.5 (78.2–99.2)	66.7 (51.6–79.0)	8.61	48.8	96.6	<0.001
	NLR	0.65 (0.51–0.78)	—	—	—	—	—	0.057
	SII	0.54 (0.40–0.69)	—	—	—	—	—	0.596
PE vs. GHT	PSP	0.80 (0.66–0.93)	68.2 (47.3–83.6)	85.0 (64.0–94.8)	10.70	82.4	70.8	0.001 *
PE vs. (Ctrl + GHT)	PSP	0.82 (0.73–0.91)	68.2 (47.3–83.6)	83.9 (72.8–91.0)	10.70	60.0	88.1	<0.001
HDP (PE + GHT) vs. Ctrl	PSP	0.77 (0.67–0.86)	92.9 (81.0–97.5)	61.9 (46.8–75.0)	8.20	71.4	89.7	<0.001

Abbreviations: AUC: area under the ROC curve (95% CI by bootstrap, 2000 iterations); Sens.: sensitivity; Spec.: specificity; PPV: positive predictive value; NPV: negative predictive value; PE: preeclampsia; GHT: gestational hypertension; HDP: hypertensive disorders of pregnancy. Values shown in italics below sensitivity and specificity estimates are Wilson 95% confidence intervals (%). Because predictive values were calculated within case–control comparison sets with artificially defined disease prevalence, PPV and NPV should be interpreted descriptively and should not be extrapolated to unselected obstetric populations with different background disease prevalence. Cutoff values determined by Youden index. * *p* < 0.05 is considered statistically significant.

**Table 6:** Binary logistic regression: variables associated with preeclampsia (PE vs. Control + GHT).

Variable	β	SE	OR (95% CI)	*p* Value
PSP (ng/mL)	+0.842	0.298	2.32 (1.29–4.16)	0.005 *
Age (years)	+0.064	0.082	1.07 (0.91–1.25)	0.437
BMI (kg/m^2^)	+0.011	0.083	1.01 (0.86–1.19)	0.892
Gestational age (weeks)	−1.582	0.467	0.21 (0.08–0.51)	0.001 *

Abbreviations: β: regression coefficient; SE: standard error; OR: odds ratio; CI: confidence interval. Outcome: preeclampsia (1) vs. control + GHT (0); *n* = 84. Model calibration: Hosmer–Lemeshow χ^2^ = 4.18, df = 8, *p* = 0.840; Nagelkerke R^2^ = 0.726. * *p* < 0.05 statistically significant.

**Table 7:** Multinomial logistic regression: predictors across the spectrum of hypertensive disorders (Reference: Control).

Variable	GHT vs. Control OR (95% CI)	*p*	PE vs. Control OR (95% CI)	*p*
PSP (ng/mL)	1.42 (1.05–1.92)	0.022 *	2.99 (1.57–5.68)	0.001 *
Age (years)	1.08 (0.93–1.26)	0.293	1.14 (0.92–1.40)	0.224
BMI (kg/m^2^)	1.14 (0.97–1.35)	0.111	1.13 (0.91–1.40)	0.263
Gestational age (weeks)	0.29 (0.14–0.62)	0.001 *	0.08 (0.02–0.26)	<0.001 *

Abbreviations: OR: odds ratio; CI: confidence interval; GHT: gestational hypertension; PE: preeclampsia. Reference category: normotensive controls. McFadden Pseudo R^2^ = 0.470; LLR *p* < 0.001. * *p* < 0.05 is considered statistically significant.

## Data Availability

The data presented in this study are available on reasonable request from the corresponding author. The data are not publicly available due to ethical restrictions and patient privacy considerations.

## Supplementary Materials

The following supporting information can be downloaded at: https://www.mdpi.com/article/10.3390/medicina62071361/s1, STROBE Statement—Checklist for Case-Control Studies.

## Abbreviations

The following abbreviations are used in this manuscript: ACOG: American College of Obstetricians and Gynecologists; AISI: aggregate index of systemic inflammation; ANOVA: analysis of variance; AUC: area under the receiver operating characteristic curve; BMI: body mass index; BP: blood pressure; CBC: complete blood count; CI: confidence interval; COVID-19: coronavirus disease 2019; CRP: C-reactive protein; CV: coefficient of variation; DBP: diastolic blood pressure; DNA: deoxyribonucleic acid; ELISA: enzyme-linked immunosorbent assay; GDM: gestational diabetes mellitus; GHT: gestational hypertension; HDP: hypertensive disorders of pregnancy; HELLP: hemolysis, elevated liver enzymes, and low platelets; HMGB1: high mobility group box 1; IL-6: interleukin-6; IQR: interquartile range; ISSHP: International Society for the Study of Hypertension in Pregnancy; LLR: log-likelihood ratio; MLR: monocyte-to-lymphocyte ratio; NLR: neutrophil-to-lymphocyte ratio; NLRP3: NLR family pyrin domain containing 3; NPV: negative predictive value; OR: odds ratio; PE: preeclampsia; PIV: pan-immune-inflammation value; PlGF: placental growth factor; PLR: platelet-to-lymphocyte ratio; PPROM: preterm premature rupture of membranes; PPV: positive predictive value; PSP: pancreatic stone protein; REG1A: regenerating islet-derived protein 1 alpha; ROC: receiver operating characteristic; ROS: reactive oxygen species; SBP: systolic blood pressure; SD: standard deviation; SE: standard error; sFlt-1: soluble fms-like tyrosine kinase-1; SII: systemic immune-inflammation index; SIRI: systemic inflammation response index; STARD: Standards for Reporting Diagnostic accuracy studies; STROBE: Strengthening the Reporting of Observational Studies in Epidemiology; TLR-4: Toll-like receptor 4; TNF-α: tumor necrosis factor alpha; WBC: white blood cell count.
