# Supplementary material for: Serum Pancreatic Stone Protein Across the Spectrum of Hypertensive Disorders of Pregnancy: Discrimination of Preeclampsia Compared with Conventional Inflammatory Indices
Source: Medicina (Kaunas). 2026 Jul 15;62(7):1361. doi: 10.3390/medicina62071361 (PMC13413923; doi:10.3390/medicina62071361)
Supplement: Supplementary file 1 [file medicina-62-01361-s001.zip › medicina-4366605-supplementary.pdf]

STROBE Statement — Checklist for Case-Control Studies

Manuscript:

Serum Pancreatic Stone Protein Across the Spectrum of Hypertensive Disorders of Pregnancy: Discrimination of Preeclampsia Compared with Conventional Inflammatory Indices

This checklist documents how the present manuscript adheres to the STROBE (Strengthening the Reporting of Observational Studies in Epidemiology) statement for the reporting of case-control studies. Each of the 22 STROBE items is mapped to its corresponding location in the manuscript.

Item No	Recommendation	Location in Manuscript
**Title and abstract**		
1	(a) Indicate the study's design with a commonly used term in the title or the abstract. (b) Provide in the abstract an informative and balanced summary of what was done and what was found.	(a) Title: "Serum Pancreatic Stone Protein Across the Spectrum of Hypertensive Disorders of Pregnancy: Discrimination of Preeclampsia Beyond Conventional Inflammatory Indices" — "prospective case-control" specified in Methods 2.1.<br>(b) Abstract — structured summary (Objective, Methods, Results, Conclusions).
**Introduction**		
2	Background/rationale: Explain the scientific background and rationale for the investigation being reported.	Section 1 (Introduction), paragraphs 1–6 — epidemiology of HDP, pathophysiology of PE, role of CBC-derived inflammatory indices, biology of PSP, prior PSP-pregnancy data.
3	Objectives: State specific objectives, including any prespecified hypotheses.	Section 1 (Introduction), final paragraph — primary objective (compare PSP across control/GHT/PE), secondary objectives, and explicit hypothesis statement.
**Methods**		
4	Study design: Present key elements of study design early in the paper.	Section 2.1 (Study Design and Setting) — "prospective case-control study" stated in the first sentence.
5	Setting: Describe the setting, locations, and relevant dates, including periods of recruitment, exposure, follow-up, and data collection.	Section 2.1 — Department of Obstetrics and Gynecology, Ankara Etlik City Hospital; recruitment 5 March – 30 April 2026; ethics approval date 3 March 2026.
6	Participants: (a) Give the eligibility criteria, and the sources and methods of case ascertainment and control selection. Give the rationale for the choice of cases and controls. (b) For matched studies, give matching criteria and the number of controls per case.	(a) Section 2.2 (Participants) — ACOG 2020 criteria for PE/GHT diagnosis; consecutive eligible women presenting during the predefined enrolment period (5 March – 30 April 2026); eligibility, inclusion and exclusion criteria detailed.<br>(b) Not a matched design; rationale provided in Discussion (gestational-age-matched controls were not feasible — acknowledged as a limitation).
7	Variables: Clearly define all outcomes, exposures, predictors, potential confounders, and effect modifiers. Give diagnostic criteria, if applicable.	Section 2.2 (Participants) — group definitions and ACOG 2020 PE diagnostic criteria; Section 2.4 (Inflammatory Index Calculation) — formulas for NLR, PLR, MLR, SII, SIRI, PIV, AISI; Section 2.6 (Statistical Analysis) — confounders considered (age, BMI, gestational age).
8	Data sources/measurement: For each variable of interest, give sources of data and details of methods of	Section 2.3 (Sample Collection and PSP Measurement) — sandwich ELISA (Bioassay Technology Laboratory, E6073Hu), duplicate measurements with mean used for

Item No	Recommendation	Location in Manuscript
	assessment (measurement). Describe comparability of assessment methods if there is more than one group.	analysis, intra-assay CV < 8%, inter-assay CV < 10%; samples stored at −80 °C with storage duration of approximately two weeks to two months and a single freeze-thaw cycle; laboratory personnel blinded to clinical group assignment; CBC parameters obtained from automated hematology analyzer; assessment methods identical across all three groups.
**9**	Bias: Describe any efforts to address potential sources of bias.	Section 2.6 (Statistical Analysis) — Bonferroni correction for multiple testing; Section 2.5 (Power Analysis) — a priori sample-size estimation; Section 4 (Discussion, Limitations) — explicit acknowledgement of selection bias from single-center recruitment and gestational-age confounding.
**10**	Study size: Explain how the study size was arrived at.	Section 2.5 (Power Analysis and Sample Size) — a priori target of 31/group based on Brun et al. 2023 effect size; achieved sizes (Control n = 42, GHT n = 20, PE n = 22) reflect natural admission pattern at the tertiary center.
**11**	Quantitative variables: Explain how quantitative variables were handled in the analyses. If applicable, describe which groupings were chosen and why.	Section 2.6 (Statistical Analysis) — Shapiro-Wilk normality testing; ANOVA / Kruskal-Wallis for three-group comparisons; t-test / Mann-Whitney U with Bonferroni correction for pairwise comparisons; ROC analysis for discriminatory performance with Youden-index cutoffs and Wilson 95% confidence intervals for sensitivity and specificity (Table 5); logistic and multinomial regression for adjusted associations; reporting of diagnostic-accuracy components guided by the STARD 2015 statement.
**12**	Statistical methods: (a) Describe all statistical methods, including those used to control for confounding. (b) Describe any methods used to examine subgroups and interactions. (c) Explain how missing data were addressed. (d) If applicable, explain how matching of cases and controls was addressed. (e) Describe any sensitivity analyses.	(a) Section 2.6 — binary logistic and multinomial logistic regression with adjustment for age, BMI, and gestational age.<br>(b) Section 3 (Results) — within-PE post-hoc subgroup analysis (preterm vs. term).<br>(c) No missing data — Results 3.1 states explicitly that complete laboratory and clinical data were available for all 84 participants with no missing values for any analyzed variable.<br>(d) Not a matched design.<br>(e) Section 2.5 — sensitivity analysis confirming detectable effect sizes at achieved n.
**Results**		
**13**	Participants: (a) Report numbers of individuals at each stage of the study. (b) Give reasons for non-participation at each stage. (c) Consider use of a flow diagram.	(a) Section 3.1 (Results) — 84 participants enrolled (Control 42, GHT 20, PE 22); no exclusions during data collection.<br>(b) No non-participation reported — all eligible women who agreed to participate were enrolled.<br>(c) Flow diagram not provided as no participants were excluded post-recruitment; numbers presented in text and Table 1.
**14**	Descriptive data: (a) Give characteristics of study participants and information on exposures and potential confounders. (b) Indicate number of participants with missing data for each variable of interest.	(a) Table 1 — demographic and clinical characteristics; Table 2 — PSP and inflammatory index distributions across groups.<br>(b) No missing data; complete data for all 84 participants.

Item No	Recommendation	Location in Manuscript
**15**	Outcome data: Report numbers in each exposure category, or summary measures of exposure.	Table 2 — PSP medians (IQR), means ± SD for inflammatory indices across groups; pairwise post-hoc p-values reported.
**16**	Main results: (a) Give unadjusted estimates and, if applicable, confounder-adjusted estimates and their precision. Make clear which confounders were adjusted for and why they were included. (b) Report category boundaries when continuous variables were categorized. (c) If relevant, consider translating estimates of relative risk into absolute risk for a meaningful time period.	(a) Tables 5 (ROC) and 6 (binary logistic regression) and 7 (multinomial regression) — unadjusted (ROC AUCs) and adjusted (OR with 95% CI) estimates; adjustment for age, BMI, gestational age justified in Section 2.6 and Discussion.<br>(b) Cutoffs (8.61, 10.70, 8.20 ng/mL) reported in Table 5 with rationale (Youden index).<br>(c) Not applicable (cross-sectional outcome assessment).
**17**	Other analyses: Report other analyses done—e.g., analyses of subgroups and interactions, and sensitivity analyses.	Section 3 — within-PE preterm vs. term post-hoc analysis (p = 0.109); Spearman correlations between PSP and inflammatory indices/clinical parameters (Table 4); sensitivity discussion in Section 2.5 and Discussion (Limitations).
**Discussion**		
**18**	Key results: Summarise key results with reference to study objectives.	Section 4 (Discussion), opening paragraph — graded PSP elevation across HDP spectrum, ROC AUCs for all four scenarios, comparative performance vs. CBC-derived inflammatory indices.
**19**	Limitations: Discuss limitations of the study, taking into account sources of potential bias or imprecision. Discuss both direction and magnitude of any potential bias.	Section 4 (Discussion), Limitations subsection — modest sample sizes, gestational-age mismatch between groups, single-center two-month recruitment window with potential selection bias, exploratory nature of regression and ROC-derived cutoffs, lack of angiogenic biomarker comparison; bias direction discussed for each.
**20**	Interpretation: Give a cautious overall interpretation of results considering objectives, limitations, multiplicity of analyses, results from similar studies, and other relevant evidence.	Section 4 (Discussion) — interpretation as biomarker reflecting graded HDP severity; mechanistic placenta–pancreas axis hypothesis; comparison with Brun et al. 2023 and Vonzun et al. 2023; explicit cautions about exploratory framework; convergent evidence from Polat et al. 2026 [28] cited as independent supporting observation.
**21**	Generalisability: Discuss the generalisability (external validity) of the study results.	Section 4 (Discussion), Limitations subsection — explicit statement that PSP cutoff values may not be directly generalizable to other geographic regions or populations with different demographic characteristics and baseline disease prevalences.
**Other information**		
**22**	Funding: Give the source of funding and the role of the funders for the present study and, if applicable, for the original study on which the present article is based.	Funding section (after Conclusions) — “This research received no external funding.”

Reference: von Elm E, Altman DG, Egger M, et al. The Strengthening the Reporting of Observational Studies in Epidemiology (STROBE) statement: guidelines for reporting observational studies. J Clin Epidemiol. 2008;61(4):344-349. doi:10.1016/j.jclinepi.2007.11.008
